# Modulation of Immune Cell Functions by the E3 Ligase Cbl-b

**DOI:** 10.3389/fonc.2015.00058

**Published:** 2015-03-11

**Authors:** Christina Lutz-Nicoladoni, Dominik Wolf, Sieghart Sopper

**Affiliations:** ^1^Department of Hematology and Oncology, Medical University Innsbruck, Innsbruck, Austria; ^2^Tumor Immunology Laboratory, Tyrolean Cancer Research Institute, Innsbruck, Austria; ^3^Medical Clinic III for Oncology, Haematology and Rheumatology, University Clinic Bonn (UKB), Bonn, Germany

**Keywords:** cancer, autoimmunity, immunotherapy, ubiquitination, adoptive cell transfer

## Abstract

Maintenance of immunological tolerance is a critical hallmark of the immune system. Several signaling checkpoints necessary to balance activating and inhibitory input to immune cells have been described so far, among which the E3 ligase Cbl-b appears to be a central player. Cbl-b is expressed in all leukocyte subsets and regulates several signaling pathways in T cells, NK cells, B cells, and different types of myeloid cells. In most cases, Cbl-b negatively regulates activation signals through antigen or pattern recognition receptors and co-stimulatory molecules. In line with this function, *cblb*-deficient immune cells display lower activation thresholds and *cblb* knockout mice spontaneously develop autoimmunity and are highly susceptible to experimental autoimmunity. Interestingly, genetic association studies link CBLB-polymorphisms with autoimmunity also in humans. Vice versa, the increased activation potential of *cblb*-deficient cells renders them more potent to fight against malignancies or infections. Accordingly, several reports have shown that *cblb* knockout mice reject tumors, which mainly depends on cytotoxic T and NK cells. Thus, targeting Cbl-b may be an interesting strategy to enhance anti-cancer immunity. In this review, we summarize the findings on the molecular function of Cbl-b in different cell types and illustrate the potential of Cbl-b as target for immunomodulatory therapies.

## Modulation of Immune Cell Functions by the E3 Ligase Cbl-b

Ubiquitination represents an important post-transcriptional regulatory mechanism of various cellular pathways. The specificity of this process is conferred by E3 ubiquitin ligases. Over the last decade, many E3 ubiquitin ligases involved in the fine-tuning of immunological responses have been described (1–3). Among these, Casitas B-lineage lymphoma proto-oncogene-b (Cbl-b) stands out as one of the most important gate keepers of immune activation due to its function as a non-redundant negative regulator of immune activation (4–7). Here, we report on the recent advances in our understanding of the molecular function of Cbl-b and discuss potential clinical implications of Cbl-b targeting for cancer immunotherapy.

## Protein Ubiquitination

Post-transcriptional protein modification such as ubiquitination adds an additional important layer to the regulation of cellular processes. Ubiquitin (Ub) is a 76 amino acid peptide, which is tagged to proteins through a process involving three enzymes: E1, E2, and E3. Ubiquitination is initiated by an ubiquitin activating enzyme (E1) forming a thiol ester bond with the C-terminal glycine residue of ubiquitin. The activated Ub can then be transferred to one of the Ub conjugating enzymes (E2) via a similar thioester linkage. In a final step, E3 ligases catalyze the formation of an isopeptide bond between Ub and a specific lysine residue of the target protein (8, 9). In addition to 2 E1 and 35 E2 ligases, the mammalian genome encodes over 1000 E3 proteins, which are responsible for the broad range of target proteins regulated by ubiquitin modification (10).

Poly ubiquitination via lysine residue K48 serves as a proteasome-targeting signal leading to degradation of the substrate protein via the 26S proteasome while mono-ubiquitination or poly ubiquitination via other lysine residues, e.g., K63, mainly modifies protein function by altered protein trafficking and subcellular localization (11, 12). Cytoplasmic signaling proteins and nuclear transcription factors tend to be ubiquitinated for proteasomal degradation while surface receptors like receptor tyrosine kinases, G-protein-coupled receptors, and the TCR are more often regulated by endocytosis and subsequent lysosomal degradation (13–15).

## Molecular Mechanisms of Cbl-Family E3 Ligases

**Figure 1:**
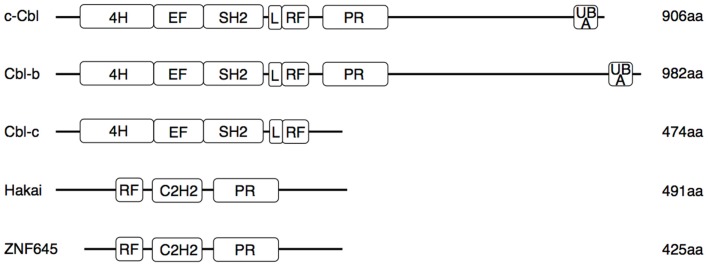
**Functional domains of human Cbl and Cbl-like proteins**. Three N-terminal regions [4-helical bundle (4H), EF-hand domain, and SH2 domain] constitute the tyrosine kinase binding (TKB) domain. Separated by a conserved linker (L) region follows a RING finger (RF) domain, which binds to E2 enzymes. The proline-rich sequence (PR) interacts with SH3 domains of various proteins involved in signaling and endocytosis. In the Cbl-like proteins, the order of the RF and TKB (here comprising a single C3H2 domain) is reversed. Only c-Cbl and Cbl-b contain an ubiquitin-associated domain (UBA) involved in binding and dimerization of ubiquitin. Several tyrosine residues in the L region and in the less conserved C-terminal half can be phosphorylated, resulting in conformational changes necessary for the ligase activity or interaction with SH2 domains of signaling proteins. Mutations associated with myeloproliferative disorders are located in the RF and L regions.

The Cbl proteins are encoded by a highly conserved gene family, which can be found from nematodes to mammals (16). The name is derived from the retroviral oncoprotein v-Cbl promoting development of B cell leukemia in mice. V-Cbl represents a dominant mutant antagonizing the function of its cellular homolog c-Cbl (17, 18). The mammalian genome encodes three Cbl proteins (Figure 1): c-Cbl (also termed Cbl2, Cbl-SL, or RNF55) (18), Cbl-b (also termed RNF56) (19), and Cbl-c (also called Cbl-3) (20, 21). While c-Cbl and Cbl-b are expressed in a wide range of tissues, expression of Cbl-3 and another structurally similar E3 ligase, Cbl-like protein-1 (CBLL1, also called HAKAI) is restricted to epithelia (17, 19–23). Recently, ZNF645, a new E3 ligase with structural homologies to Cbl proteins, has been described as testis specific gene (24). Cbl proteins interact with target proteins via their protein–protein interaction domains (Figure 1), allowing regulation of multiple pathways (25). This interaction involves recognition of specific phosphotyrosine-containing sequence motifs that are generated on protein tyrosine kinases (PTK). Thus, the current paradigm holds that Cbl-family E3 ligases are selective regulators of activated PTKs (26).

**Figure 2:**
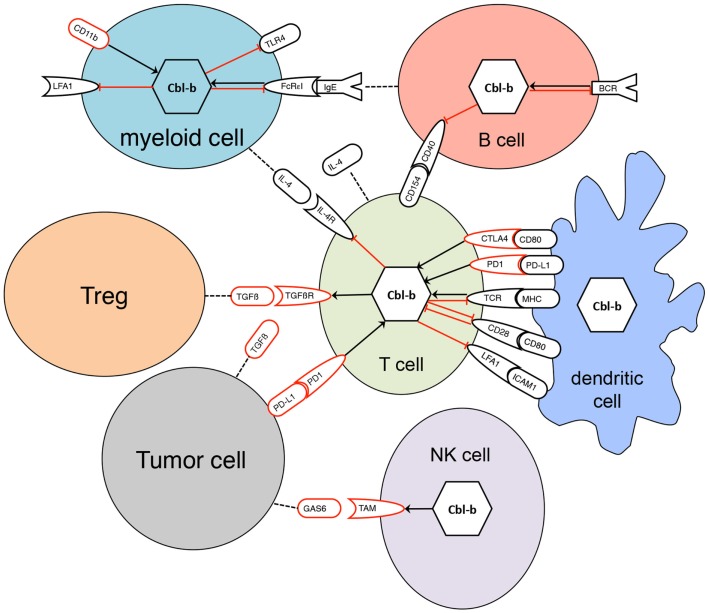
**Interaction of Cbl-b with signaling pathways in diverse cells**. Black receptors represent activating signaling pathways, red receptors inhibitory pathways. Black arrows indicate positive regulation, red bar-headed lines are representative for negative regulation. Dotted lines indicate secretion of proteins. Regulation of Cbl-b occurs not only at the transcriptional level but also by post-transcriptional mechanisms such as phosphorylation, degradation, or sequestration to specific protein complexes.

All three mammalian Cbl proteins are RING-type E3 ligases containing an N-terminal tyrosine kinase binding (TKB) domain consisting of a four-helical bundle, a calcium binding EF-hand and a Src homology (SH2) domain, followed by a linker helical region and the RING domain (Figure 1), responsible for their catalytic function (16, 27–32). The evolutionary highly conserved N-terminal RING-type zinc finger domains forming the basic E3 module of Cbl proteins are essential and sufficient for activated PTK directed E3 ligase activity (33, 34) as evidenced by a loss of function mutation in the E3 ligase domain of *cblb*, which phenocopies the *cblb* knockout phenotype *in vivo* (35). Additionally, c-Cbl and Cbl-b contain proline-rich regions mediating the association with tyrosine- and serine phosphorylation sites, and an ubiquitin-associated (UBA)/leucine zipper domain for dimerization (Figure 1) (16, 36–38). Via their protein interaction domains Cbl proteins interact with a large number of target proteins either as E3 ligases or adaptor molecules, e.g., with Src family kinases, SH2-domain containing proteins of the PTK-dependent signaling network including Vav guanine exchange factors, the p85 subunit of phosphatidylinositol 3-kinase (PI3K), and adaptor proteins of the Crk-family allowing the regulation of multiple pathways (26). Proteins ubiquitinated by Cbl proteins are either degraded in the proteasome or sequestered to specific cellular locations. Of the three Cbl proteins in mammals, Cbl-b is preferentially expressed in peripheral lymphoid organs suggesting a prominent function for adaptive immune responses. Specifically, Cbl-b seems to be central for maintenance of peripheral tolerance as *cblb* knock out mice develop spontaneous autoimmunity characterized by auto-antibody production and infiltration of activated T and B cells into multiple organs (4, 5). Cbl-linked networks (Figure 2) have been implicated in the control of the immune system, cell proliferation, differentiation, and cell morphology (25, 39). Spatial or temporal dysregulation of Cbl proteins results in autoimmunity or increased tumor progression.

## Cbl-b Function in T Cells

Cbl-b is highly expressed in murine and human CD4^+^ and CD8^+^ T cells (GFN SymAtlas, http://biogps.org) and its expression levels are tightly regulated by CD28 and CTLA-4 stimulation (40, 41) and other co-stimulatory and inhibitory signals (Figure 2) (42). Over the years, work by several groups has documented an essential role of Cbl-b in the negative regulation of T cell activation (6, 7, 39).

T cell activation and tolerance induction are tightly controlled processes regulating immune responses to pathogens and tumors while preventing autoimmunity. Autoimmunity is mainly averted through central tolerance by negative selection of thymocytes carrying TCR for self-antigens (43, 44). However, mechanisms of peripheral tolerance are needed for T cells that escaped thymic selection, and include tolerance of low level expressed or low-affinity antigens, immunosuppression mediated by regulatory T cells (Treg), and other suppressive cell populations, induction of anergy, e.g., in the absence of co-stimulation and activation-induced cell death (43, 45, 46). While maintaining tolerance prevents autoimmunity on one hand, tumor induced anergy induction of T cells is critical and hazardous on the other hand. Immunosuppression by cancer cells involves induction and expansion of various immunosuppressive cell types such as Tregs and myeloid derived suppressor cells as well as production of inhibiting cytokines, e.g., transforming growth factor-beta (TGF-β), and finally supports tumor cells to escape the immune system (47).

T cells require two signals for proper activation: the first provided by interaction of the TCR complex with the cognate peptide antigen presented by major histocompatibility complex molecules and a second through co-stimulatory molecules on antigen presenting cells. Following initial triggering of the antigen receptor, the Src family kinases Lck and Fyn are recruited to phosphorylate ZAP-70, which subsequently phosphorylates SLP-76 and LAT. Subsequently, a multi-subunit protein complex containing inducible T cell kinase, PI3K, phospholipase C-γ (PLCγ), and Vav1 is formed resulting in PLCγ-regulated calcium influx, cytoskeletal rearrangements via Vav1, Rac, WASP, and activation of protein kinase C-theta (PKCθ) (48). PKCθ is a key molecule of the immunological synapse responsible for cytokine responses, proliferation of T cells (49), and T_H_17-mediated autoimmunity (50). The sustained calcium influx following TCR engagement activates the phosphatase calcineurin, which subsequently dephosphorylates the NFAT family of transcription factors. Dephosphorylated NFAT proteins then translocate to the nucleus to induce expression of various genes (51).

Final activation of T cells is achieved by a second signal delivered by several co-stimulatory molecules, of which CD28 is the most prominent. CD28 is activated by binding to its ligands CD80 or CD86 to enhance TCR proximal signaling and to induce additional pathways, leading to activation of NF-κB, NFAT, and AP-1 transcription factors (52). Cooperation of NFAT with AP-1 is required for IL-2 gene transcription and mRNA stability (51). Furthermore, CD28 co-stimulation leads to cytoskeletal reshuffle in order to form and stabilize the immunological synapse. Strong TCR stimulation without co-stimulation or TCR stimulation by a low-affinity antigen results in T cell anergy instead of activation (53) and anergic T cells remain in a viable, but unresponsive state.

After receiving tolerizing signals, e.g., TCR stimulation in the absence of co-stimulation, Cbl-b is up-regulated (40, 41) and inhibits signaling events downstream of PI3K. In Jurkat cells, this was mediated by ubiquitination of the p85 subunit of PI3K leading to reduced interaction of p85 with CD28 (54). However, ubiquitination of p85 was never shown in primary cells, and these findings have recently been challenged by a report that Cbl-b does not directly inhibit PI3K, but suppresses PTEN inactivation independently of its ubiquitin ligase activity (55). Cbl-b associates with Nedd4 inhibiting its autophosphorylation and subsequent ubiquitination of PTEN, thereby inhibiting down-regulation of PTEN. In addition, introducing *nedd4* deficiency into *cblb-*deficient mice abrogates the hyperresponsive phenotype of *cblb^−^*^/−^ T cells. As these experiments have been exclusively performed in murine models, it is still possible that direct effects of Cbl-b on PI3K contribute to the modulation of CD28 signaling in human T cells.

Furthermore, Cbl-b reduces phosphorylation and activation of PLCγ-1 in anergic cells. Accordingly, loss of Cbl-b rescues reduced calcium mobilization of anergic T cells, resulting in impaired induction of T cell tolerance (56). Cbl-b regulates calcium influx and IL-2 production through Vav-1 activation (57). Cbl-b directly interacts with Vav1 through its N-terminal proline-rich region, thereby controlling phosphorylation of Vav1, but not by targeting it for degradation. Whereas in wild-type cells TCR plus CD28 coreceptor stimulation is required for Vav1 activation, in *cblb-*deficient cells sole TCR stimulation is sufficient for Vav1 activation (57), which is important for formation and stability of the immunologic synapse. Cbl-b and c-Cbl together promote antigen-induced down-regulation of the TCR (58). Moreover, Cbl-b was shown to repress NF-κB transactivation (59). Following stimulation of CD28, Cbl-b is post-transcriptionally eliminated by ubiquitination and proteasomal degradation, which is a prerequisite for the maintenance of T cell proliferation and IL-2 production (41). Gruber et al. described an antagonistic cross-talk between PKCθ and Cbl-b, which colocalize upon TCR and CD28 co-stimulation, resulting in phosphorylation of Cbl-b by PKCθ probably inducing a conformational change of Cbl-b (60). Subsequently, Cbl-b is ubiquitinated by the E3 ligase Nedd4 mediating proteasomal degradation (61). In summary, genetic loss of Cbl-b in naive T cells uncouples TCR stimulation from the requirement of CD28 co-stimulation for effective proliferation and IL-2 secretion. *Cblb*-deficient T cells are hyperresponsive, and even upon TCR stimulation in the absence of CD28 co-stimulation they proliferate comparable to wild-type T cells after double stimulation with anti-CD3 and anti-CD28 antibodies (4, 5).

Cbl-b is also involved in activation-induced cell death. *Cblb*-deficient CD4^+^ T_H_1 cells are resistant to apoptosis induction by CD3 stimulation in the absence of CD28^+^ co-stimulation (62) and also clonal deletion of CD8^+^ T cells was shown to depend on Cbl-b (63, 64). This is important in the context of chronic viral infection where virus-specific CD8^+^ T cells become exhausted. It was shown that functional impairment (exhaustion) and to a lesser extent also elimination of virus-specific T cell clones was delayed in Cbl-b deficient animals persistently infected with LCMV leading to immunopathology and decreased survival (63).

The central role of Cbl-b in the maintenance of the balance between T cell tolerance and T cell activation is mirrored in the autoimmune phenotype of *cblb* knock out mice. Despite normal T cell development *cblb* knock out mice are highly susceptible to experimental autoimmune encephalomyelitis (5). Furthermore, depending on the genetic background *cblb-*deficient mice develop spontaneous autoimmunity characterized by auto-antibody production, infiltration of activated T and B lymphocytes into multiple organs, increased size of submandibular gland, and parenchymal damage starting at 6 months of age (4). Along this line, increased collagen-induced autoimmune arthritis induced by immunization and subsequent boosting with collagen is found in *cblb* knock out DBA/1 mice compared to wildtype (wt) DBA/1 mice (56). In another model of anergy induction in T cells p14/TCRVaVb8.2 transgenic mice recognizing the LCMV p33 peptide presented by MHC class I were injected with the cognate p33 peptide. On a *cblb*-deficient background increased *in vivo* expansion of antigen-specific T cells was detected, and instead of anergy the rechallenge with p33 induced a cytokine storm followed by massive edemas in multiple organs and high mortality of the animals (56).

Cbl-b controls the threshold for T cell activation (40, 41) and regulates the affinity of antigens to their TCR (65). Gronski et al. demonstrated increased development of autoimmune diabetes by crossing p14/Rip-gp transgenic mice onto a *cblb^−^*^/−^ background. About 100% of *cblb*-deficient p14/Rip-gp mice versus <50% of *cblb*^+/+^ p14/Rip-gp mice developed diabetes when infected with LCMV expressing the low-affinity ligand LF6, accompanied by enhanced T cell proliferation and enhanced cytotoxic T-lymphocyte function in *cblb*-deficient mice (65).

Transforming growth factor-β plays a central role in T cell differentiation (66) by inhibiting T_H_1 and T_H_2 polarization (67) and triggering differentiation of inducible Tregs or, in cooperation with IL-6, induction of T_H_17 cells (68). Additionally, TGF-β exerts immune suppressive functions and inhibits T cell activity averting development of autoimmunity (69). TGF-β secretion by tumor cells and tumor-infiltrating Tregs therefore contributes to immune evasion of tumor cells (70). Cbl-b deficiency renders peripheral CD4^+^ and CD8^+^ T cells partially resistant to inhibitory effects exerted by Tregs via disruption of the TGF-β pathway (56, 71–76). The interaction of CD4^+^CD25^+^ regulatory T cells and CD4^+^CD25*^−^* effector cells in *cblb^−^*^/−^ mice was extensively studied by Wohlfert et al. (75), demonstrating normal function of *cblb*-deficient Tregs, but resistance of cbl-b deficient CD4^+^CD25*^−^* T cells to inhibition by either Tregs or soluble TGF-β. In the presence of TGF-β, naive CD4^+^CD25^+^ T cells can be converted into CD4^+^CD25^+^FoxP3^+^ inducible Tregs (77). This differentiation is negatively regulated by the PI3K/Akt/mTOR and the FOXO1/3a pathway (78, 79). Cbl-b promotes TGF-β-mediated iTreg conversion by tuning the threshold of T cell activation via an Akt2-dependent mechanism (80). Recently, SMAD7, a negative regulator of TGF-β receptor signaling, was identified as a critical target of Cbl-b (81), thus regulating sensitivity toward TGF-β effects. On a molecular level, Cbl-b directly interacts with and ubiquitinates SMAD7, targeting it for degradation and thereby increasing TGF-β/SMAD signaling in T cells. Accordingly, resistance of *cblb-*deficient T cells to TGF-β is abrogated by conditional deletion of SMAD7 (81).

Moreover, Cbl-b seems to be involved in the negative regulation of CD8^+^ T cells via the interaction of programed cell death ligand 1 (PD-L1) on dendritic cells (DCs) programed cell death 1 (PD1). It was recently shown that PD-L1 silencing in DC blocks up-regulation of Cbl-b in CD8^+^ T cells after antigen presentation resulting in hyperactive pro-inflammatory TCR^high^ CD8^+^ T cells and accelerated anti-tumor immune responses in the EG.7 mouse tumor model (42). Regulation of Cbl-b by PD1 possibly contributes to the clinical benefit seen in patients with advanced cancer after treatment with antibodies blocking the interaction between PD1 and PD-L1 (82, 83). Similarly, the improved survival after treatment of melanoma patients with CTLA-4 blocking antibodies (84) may also involve Cbl-b down-regulation as activation of CTLA-4 increases Cbl-b levels by transcriptional regulation (Figure 2) (40).

Recent evidence suggests that Cbl-b is also involved in the polarization of T helper cells into functionally distinct subsets. Levels of Cbl-b are significantly lower in T_H_2 and T_H_9 cells compared to T_H_1 and T_H_17 cells, and *in vitro* differentiation of naïve T cells from *cblb* knock out mice yielded more T_H_2 and T_H_9 cells in comparison to those derived from wt mice (85). In addition, absence of Cbl-b resulted in stronger T_H_2 and T_H_9 responses and was associated with severe airway inflammation in a mouse model of asthma (85). This, however, is somewhat in contrast to a previous report where, using a different mouse strain and different immunization protocols, increased susceptibility to asthma induction in *cblb*-deficient mice was mediated by a T_H_1 response in the lung (86). IL-4 signaling through the JAKs/Stat6 pathway is essential for both T_H_2 and T_H_9 differentiation (87). Cbl-b was shown to bind to Stat6 via its TKB domain leading to ubiquitination and degradation though the proteasome, explaining increased signaling through the Stat6 pathway in *cblb^−^*^/−^ cells, and finally increased T_H_2 differentiation (85). The molecular mechanism of increased T_H_9 differentiation, however, remains completely unclear, especially because T_H_9 cells require an active TGF-β signal to be differentiated, which, however, is blocked due to the accumulation of the inhibitory SMAD7, mentioned above.

## Targeting Cbl-b in T Cells for Tumor Immunotherapy

As already shown for various other checkpoint regulators, genetic loss of the Cbl-b protein leads to autoimmunity but is linked to improved tumor immune surveillance. A recent report using a shRNA screen for major immune regulators governing anti-cancer immune responses supports the importance of Cbl-b as checkpoint molecule (88). Along this line *cblb*-deficient animals reject tumors in various autochthonous and transplanted tumor models (4, 5). Moreover, mice carrying a *cblb* E3 ligase-defective mutation are resistant to tumor formation *in vivo* and reject experimental and spontaneous malignancies (35, 72, 73). As demonstrated by several groups, including our own, this effect is mainly mediated by CD8^+^ cytotoxic T cells and NK cells (73, 89).

In more detail, TC-1 cells, c-H-ras-transformed syngenic fibroblasts expressing the human papilloma virus 16-derived oncoproteins E6 and E7 were subcutaneously injected into wild-type, heterozygous, and *cblb* knockout C57BL/6 mice (73). Tumor growth and histology of tumors were unaltered between the groups during the first 2 weeks after tumor cell inoculation, however, after 2 weeks *cblb-*deficient recipients started to reject tumors spontaneously and tumor masses were continuously reduced until tumors even became undetectable in some animals. This strong anti-tumor response was paralleled by an increased tumor infiltration rate of CD8^+^ T cells while numbers of tumor-infiltrating CD4^+^ T cells were unaltered. Moreover, increased numbers of INF-γ secreting CD8^+^ T cells could be detected in the draining lymph nodes. Treg cells infiltrated TC-1 tumors in both *cblb*^+/+^ and *cblb^−^*^/−^ mice; however, loss of Cbl-b dramatically changed the ratio of CD8^+^ T cells to Tregs, which is also a strong predictor of improved outcome in human cancer (90). These findings are supported by the work of Chiang et al. (72), who demonstrated that *cblb*-deficient mice efficiently rejected subcutaneously inoculated EL4 and EG7 lymphoma cells lacking B7 co-stimulatory molecules. EL4 T linage lymphoma cells represent a tumor model with weak immunogenicity as well as reported TGF-β secretion, while EG7 cells are highly immunogenic EL4 transfectants expressing chicken ovalbumin (OVA) as model tumor antigen. Tumor growth of both cell lines was dramatically reduced in *cblb*-deficient animals demonstrating that genetic ablation of Cbl-b provokes the ability to reject or attenuate growths of weak and highly immunogenic tumors independent of CD28-co-stimulation of effector cells. Furthermore, when crossing ataxia telangiectasia mutated (ATM) deficient mice onto a *cblb*-deficient background, 50% of ATM*^−^*^/−^ *cblb*^+/+^ mice died within 6 months of age of spontaneous T cell lymphomas while no death was observed among ATM*^−^*^/−^ *cblb^−^*^/−^ double knockout mice. Further studies using Cbl-b E3 ligase-defective (C373A^ki/Ki^) knock in mice (91) revealed that selective genetic inactivation of Cbl-b ligase activity phenocopies the total loss of Cbl-b, resulting in hyperactivation of T cells, impaired induction of T cell anergy, spontaneous autoimmunity, and tumor rejection demonstrating that the catalytic function of Cbl-b is essential for negative regulation of T cells *in vivo* (35).

The observed increased anti-tumor response seems to be in part the result of TGF-β insensitivity due to SMAD7 degradation after Cbl-b ubiquitination. To evaluate the *in vivo* relevance of Cbl-b in SMAD7 regulation, *cblb* knockout mice were crossed with CD4Cre-*smad*7^fl/fl^ knockout mice to generate T cell-specific *cblb/smad7* double knockout animals, which were investigated concerning their *in vivo* anti-tumor responses (81). While subcutaneously injected TC-1 tumors are efficiently rejected in *cblb* single knockout mice, they substantially grow in T cell specific double knockout mice that display comparable survival rates to wildtype animals indicating that concomitant loss of *smad7* abrogates the survival benefit of *cblb*-deficient mice. This insensitivity of *cblb*-deficient T cells to negative cues from the tumor microenvironment contributes to their increased anti-tumor efficacy.

The above studies also investigated a possible therapeutic efficacy of adoptively transferred *cblb*-deficient T cells in tumor-bearing hosts. Immunodeficient recombination activating gene-2 (*rag2*)*^−^*^/−^ mice, lacking both T and B cells, were inoculated with TC1 tumor cells and treated with adoptive cell transfer (ACT) of CD8^+^ T cells from either wt or *cblb*-deficient donor mice. In contrast to adoptively transferred wt cells, *cblb*-deficient naive CD8^+^ T cells were able to markedly delay tumor growth (73). Nevertheless, ACT of polyclonal *cblb*-deficient CD8^+^ T cells into immunocompetent wild-type mice yielded contradictory results. While Chiang et al. showed that ACT of *cblb*-deficient CD8^+^ T cells into EG.7 tumor-bearing C57Bl/6 wt mice led to delayed tumor outgrowth or even eradication of established tumors (72), we found that sole ACT of polyclonal *cblb*-deficient CD8^+^ T cells in immunocompetent wildtype mice challenged with either EG.7 lymphoma or B16.ova melanoma, was not sufficient to significantly delay tumor growth (74). In contrast to other reports (92–95), we did not transfer TCR transgenic tumor-antigen specific but polyclonal T cells. Moreover, we used immunocompetent wildtype recipient mice and did not combine the ACT with induction of lymphopenia as often approached (92). As monotherapy with *cblb*-deficient polyclonal CD8^+^ T cells did not provoke any benefit in neither B16.ova melanoma nor EG.7 lymphoma injected immunocompetent mice in our hands, we established a combination therapy of ACT of *cblb-*deficient CD8^+^ T cells and DC vaccination, resulting in delayed tumor outgrowth and significantly prolonged survival rates when DC vaccination was combined with ACT of *cblb-*deficient instead of wt CD8^+^ T cells (74). The enhanced anti-tumor activity was accompanied by a significantly higher CD8^+^ T cell infiltration into the tumor and significantly increased numbers of tumor-antigen-positive OVA-specific CD8^+^ T cells as well as IFN-γ secreting cells in the tumor draining lymph node. Furthermore, specific killing of peptide-loaded target cells was significantly increased in B16.ova bearing mice treated with antigen-pulsed wt DCs and ACT of *cblb^−^*^/−^ CD8^+^ T cells compared to animals challenged with the DC vaccine in combination with wt CD8^+^ T cells. Vaccination with tumor antigen-pulsed DCs thereby provide a second stimulus leading to an efficient accumulation and *in vivo* selection of tumor antigen-specific CD8^+^ T cells. Our data indicate that *cblb* targeting in T cells alone is not sufficient to counteract cancer-associated immune suppression, but that reactivation of the engineered T cells by a DC vaccine induces a profound anti-tumor immune response in non-TCR-transgenic mice (74).

## Cbl-b Function in NK Cells

Cbl proteins not only regulate adaptive immune cell functions but are also critically involved in the regulation of innate lymphocyte populations, such as NK cells (89). NK cells are among the first cells to arrive at the inflamed tissue where they exert potent cytotoxic effector functions and modulate the local immune response (96). In addition, NK cells are critically involved in immunosurveillance of cancer (97). The cytotoxic effects of NK cells and their ability to secrete cytokines is regulated by a multitude of activating and inhibiting receptors, which are expressed in a variety of combinations in the different NK cell subsets (98). Initial work on the role of Cbl proteins in NK cells was restricted to c-Cbl, with early reports demonstrating phosphorylation of c-Cbl after activation of NK cells by various stimuli (99–101). It was shown that this leads to interaction with adaptor molecules of several signal transduction pathways (99, 102) but the functional consequences remained unclear until the group of Long showed that c-Cbl imposes a block, which can only be released by two strong synergistic activating signals. Signals through activating NK cell receptors lead to integration of c-Cbl into a macromolecular complex consisting of the adaptor Crk, the scaffold protein p130CAS and the guanine-nucleotide exchange factor C3G, which is critical for signaling in a wide variety of pathways. Inhibitory signals through KIR receptors inhibit formation and result in disassembly of this complex (103). Interestingly, there was no evidence that E3 ligase function of c-Cbl plays a role in this process. In follow up publications, it was shown that two synergistic activating signals, for example, through NKG2D and CD244 are needed to overcome blockade by c-Cbl (104) and that this synergistic action is mediated by a combined phosphorylation of separate tyrosine residues of the adaptor protein SLP-76 (105). On the other hand, c-Cbl is involved in a kind of negative feedback loop in the down regulation of NKG2D after prolonged exposure to one of its ligands, MICA (106).

Very recently, an elegant study involving genetic ablation of *cblb* demonstrated a prominent role also of Cbl-b for activation and effector functions of NK cells. Knockout of *cblb* resulted in increased cytotoxicity as well as higher production of IFN-γ and perforin (89). Site directed mutagenesis resulting in functional inactivation of the catalytic domain further confirmed that the E3 ligase activity is necessary for this effect. Moreover, *cblb-*deficient NK cells were able to prolong survival and reduce metastasis in several tumor models. Members of the TAM family of cell surface tyrosine kinase receptors (107) were identified as prime targets for Cbl-b-mediated ubiquitination in NK cells (Figure 2). Apparently, Cbl-b ubiquitinates all three TAM family members, Tyro-3, Axl, and Mer, and controls TAM receptor internalization, which is necessary for their functional activity. Small molecule inhibitors of TAM receptor kinases increased NK cell cytotoxicity to the same extent as seen in Cbl-b-deficient animals. Together, these data show that Cbl-b is a key regulator of NK cell effector mechanisms and that down modulating Cbl-b function or inhibiting its substrates is a promising approach in cancer immunotherapy.

## Cbl-b Function in B Cells

Production of autoantibodies is a prominent feature of *cblb^−^*^/−^ mice (4, 108) leading to the hypothesis that Cbl-b participates in negative regulation of B cell activation pathways (Figure 2). Moreover, B cell-specific ablation of both c-Cbl and Cbl-b in mice displays only slight developmental changes of B cells such as an increase in B1 cells and marginal zone B cells but causes aberrant B cell receptor (BCR) signaling and impaired anergy in mature B cells culminating in the development of a systemic lupus erythematosus like disease (109). Many of the interaction partners of Cbl-b documented for T cells such as PLCγ2, p85/PI3K, or the nucleotide exchange factor Vav are also involved in the normal signal cascade in B cells after activation of the BCR. Therefore, it is likely that they are also targeted by Cbl-b in B cells. Indeed, early work with a chicken B cell line documented direct association of Cbl-b with PLCγ2 (110). However, in contrast to *c-cbl* (111), knock down of *cblb* resulted in reduced PLCγ2 activation in chicken B cells whereas overexpression of Cbl-b in a mouse B cell line lead to increased Ca^++^ mobilization. Together with PLCγ2, Cbl-b formed a complex with BTK and the BLNK and was indispensable for sustained intracytoplasmic Ca^++^ concentrations, suggesting a positive regulation of BCR-mediated signaling by Cbl-b (110). These findings from B cell lines is in strong contrast to work with primary mature mouse B cells, where *cblb-*deficient cells display enhanced proliferative responses upon stimulation via BCR or CD40 (Figure 2) (4) and sustained phosphorylation of components of the BCR signaling complex as well as prolonged Ca^++^ fluxes (112). The regulation of B cell activation is seemingly mediated by an interaction of Cbl-b with the spleen tyrosine kinase Syk leading to its ubiquitination and possibly degradation (112). Syk is also targeted by c-Cbl (113). Another important signaling pathway regulated by Cbl-b is the interaction between CD40L on T cells and CD40 on B cells, which plays a central role in homeostatic regulation of B cell function. *Cblb*-deficient B cells display hyperproliferation and increased survival in response to CD40 stimulation (108). In addition, T cell independent antibody production is increased in *cblb^−^*^/−^ mice. Elegant experiments, where *rag^−^*^/−^ mice were reconstituted with *cblb-*deficient B cells and wt T cells, demonstrated that also T cell dependent antibody titers against the keyhole limpet hemocyanin (KLH) model antigen are increased (108). Nevertheless, reconstitution with a combination of *cblb-*deficient T and B cells further increased KLH-specific humoral immune response. In the absence of Cbl-b, IκBα and JNK were selectively hyperphosphorylated suggesting that of the different signaling pathways downstream of CD40, NF-κB seems to be specifically targeted by Cbl-b (108). TNF receptor-associated factors (TRAF) are important adaptor molecules and their recruitment to the cytoplasmic tail of CD40 is essential for CD40-mediated signal transduction. It was demonstrated that Cbl-b binds to TRAF-2, another E3 ligase leading to its ubiquitination thereby facilitating access of the negative regulator TRAF-3 (108), which results in NF-κB activation.

In human B cells, both c-Cbl and Cbl-b are constitutively associated with CIN85 forming a complex with BLNK (114). This interaction with CIN85 also promotes degradation of Syk. However, it was so far not investigated whether c-Cbl or Cbl-b is responsible for this molecular effect.

B cells not only produce antibodies but are also involved in antigen processing and presentation to T cells. Recently, it was shown that Cbl-b but not c-Cbl is required for endocytic trafficking of the BCR complex together with the captured antigen to the MHC II containing late endosomes (115). Although BCR is ubiquitinated following stimulation, this seems not to be mediated by Cbl-b as Cbl-b constructs lacking E3 ligase activity were able to reconstitute BCR sorting to late endosomes in *cblb^−^*^/−^ cells. Rather the UBA domain is required for endocytic trafficking, possibly acting as a scaffold.

Taken together, there is evidence that both c-Cbl and Cbl-b regulate the B cell activation process and thus fine-tune humoral immune response. The relative contribution of the two Cbl proteins in the various physiological and pathological settings still awaits rigorous investigation.

## Cbl-b Function in Dendritic Cells

Dendritic cells are leukocytes whose main function is to capture and process antigens and to present them to cells of the adaptive immune system in order to activate or tolerize them. Immature DCs are characterized by high endocytotic activity and sense and uptake pathogens through pattern recognition receptors (116). Antigens are then presented to T cells in the tumor draining lymphnode. In the same time, DCs up-regulate co-receptors such as CD80, CD86, and CD40, thereby enhancing their T cell stimulation capacity. This results in the differentiation and activation of antigen-specific T-lymphocytes, enabling them to exert their specific effector functions (117).

The E3 ligase c-Cbl has been identified as a modulator of DC activation (118). Toll-like receptor (TLR)-induced expression of pro-inflammatory cytokines and chemokines, such as IL-1α, IL-1β, IL-6, IL-12p70, and CXCL1/KC was enhanced in *ccbl-*deficient DCs upon LPS exposure. Up-regulation of cytokines was accompanied by enhanced NF-κB transactivation. Loss of c-Cbl did not only up-regulate expression of TLR-triggered pro-inflammatory cytokines but also increased basal expression under steady-state conditions. By overexpressing several mutants of c-Cbl in *ccbl* knockout DCs before LPS stimulation, the authors proved that the RING domain of c-Cbl is required for IL-12 inhibition. Furthermore, *in vivo* proliferation of CD4^+^ and CD8^+^ T cells was enhanced in mice vaccinated with OVA-pulsed DCs from *ccbl* negative donors compared to the group treated with wt DCs. Splenocytes from mice primed with *ccbl*-deficient DCs demonstrated higher peptide specific cytotoxicity, higher numbers of IFN-γ secreting CD4^+^ and CD8^+^ T cells, whereas IL-4 secretion was unaltered. Thus, DC deficient for *ccbl* is more potent inducers of T_H_1 polarization. To assess whether *ccbl*-deficiency in DCs would increase their ability to reject tumors, immune competent C57BL/6 wt mice were subcutaneously injected with OVA-expressing EG.7 lymphoma cells and vaccinated with either wt or *ccbl-*deficient bone marrow derived DCs (BMDCs). Tumor growth in mice treated with *ccbl*-deficient DCs was significantly delayed compared to the control groups suggesting that inhibition of c-Cbl could probably improve DC based vaccines (118).

Besides c-Cbl, DCs also highly express Cbl-b (119), which might be involved in a negative feed back loop regulating TLR signaling (120). Along this line, it was demonstrated that low-avidity outside-in signaling through CD11b negatively regulates TLR signaling in macrophages through increased Cbl-b-mediated degradation of MyD88 and TRIF. In addition, activation of Akt kinase after binding of either RANK or CD40 to their cognate ligands is dependant on Cbl-b, whereas in B cells c-Cbl is involved (121).

Since *cblb*-deficient mice are largely protected from tumor formation (72, 73) and adoptive transfer of *cblb* negative or transiently silenced CD8^+^ T cells in combination with DC vaccination delays tumor growth in several mouse models (74, 122) it was interesting to know whether loss of Cbl-b in DCs also contributes to tumor resistance of *cblb* knockout animals (119). We confirmed high expression of Cbl-b in murine BMDCs and showed that *cblb-*deficiency did not alter BMDC differentiation *in vitro*. While expression of several functionally important surface proteins on immature and LPS-stimulated DCs was unchanged between wt and *cblb* negative DCs, we found increased DEC-205 expression on *cblb* knockout cells (119). Since DEC-205 expression is associated with Treg induction (123), this could at least in part explain the increased frequency of tumor-infiltrating Treg in *cbl-b* negative mice (72). When comparing secretion of pro-inflammatory cytokines and chemokines in wt versus *cblb*-deficient BMDCs using different stimulation regimens, we found that LPS-induced TLR4 stimulation resulted in significantly increased TNF-α, IL-6, and MIP-1α secretion in *cblb* knockout DC. Similarly, TNF-α, together with IL-1α and MCP-1, was increased upon stimulation by the TLR-9 agonist CpG in *cblb* negative DCs compared to wt DCs. Both, TLR4 and TLR9 stimulation, induce a MyD88-dependent signaling pathway leading to production of pro-inflammatory cytokines and chemokines. This is in accordance with the role of Cbl-b in MyD88 degradation and subsequent suppression of MyD88 inflammatory responses (120). Stimulation with TLR3 agonists did not result in different cytokine profiles of wt and *cblb*-deficient DCs consistent with a predominantly MyD88-independent signaling by this TLR (124). While *cblb*-deficient DCs induce stronger allogeneic T cell responses, antigen-specific T cell proliferation was unaltered *in vitro* and *in vivo* (119). Increased cytokine production of *cblb* negative DCs seems to be insufficient to influence peptide-induced proliferation of OT-I tg CD8^+^ and OT-II tg CD4^+^ T cells, maybe due to the fact that OT-I and OT-II T cells proliferate even upon stimulation with very low antigen concentrations. Also, cross-presentation capacity of BMDCs was not affected by loss of Cbl-b. According to our *in vitro* findings, the anti-tumor responses in mice vaccinated with OVA-peptide-loaded DC were not different between the various genotypes (119). Therefore, and in contrast to c-Cbl targeting, silencing of Cbl-b in DC seems to be less promising for improving the T cell priming capacity of DC vaccines.

## Cbl-b Function in Myeloid/Monocytic Cells

In comparison to other cell types, less is known on the role of Cbl-b in cells of myeloid origin. In the initial description of Cbl-b, it was already shown that it is up-regulated in human myeloid cell lines upon differentiation (19), however, only recently evidence is emerging how Cbl-b controls several aspects of myeloid cell function. It is involved in negative feed back loops of TLR signaling (120) and regulates macrophage activation by fatty acids (125). *Cblb*-deficient mice display enhanced infiltration and activation of macrophages, resulting in peripheral insulin resistance (126) and the aggravated lung inflammation seen after LPS induced sepsis in *cblb-*knockout mice is at least in part mediated by increased activation of macrophages (127). In this context, it is also interesting to note that specific mutations of Cbl proteins, mainly c-Cbl and to a lesser extent also Cbl-b, have been associated with certain myeloproliferative disorders (128, 129), already pointing out to an important role of these proteins for myeloid cell biology. In addition, c-Cbl is frequently mutated in advanced mastocytosis or mast cell leukemia (130). The mutations found not only in *ccbl* but also in *cblb* are located mainly in the RING finger (RF) and linker domains, clustering in and around the binding pocket for E2 interactions, suggesting that loss of E3 ligase activity is important for the oncogenic process (131). These clinical findings are endorsed by studies using mice with hematopoietic cells deficient of both *ccbl* and *cblb*, which display rapidly fatal myeloproliferative disease (132).

However, Cbl proteins are also relevant in the non-cancer setting in myeloid cells. As an example, challenging mice with LPS lead to severe acute lung inflammation, which is more pronounced in *cblb* knockout animals (127). In this TLR4-dependent model of sepsis stimulation with LPS increased activation of neutrophils as evidenced by augmented CD11b expression and CD62L shedding. In addition, production of TNF-α and MIP-1α after LPS stimulation was higher in *cblb-*deficient animals, whereas other cytokines such as RANTES and IL-6 remained unchanged but showed Cbl-b dependent regulation when polymicrobial sepsis was induced after cecal ligation and puncture (127), or after cardiotoxin-induced muscle damage (133). Genetic ablation of *cblb*, however, had no obvious effect on cytokine production after stimulation with TLR3 agonists (127), which is in line with our observation using BMDC (119). It was also demonstrated that Cbl-b controls TLR4 signaling (Figure 2) via rapid down regulation of TLR4 and by regulating the association between TLR4 and the adaptor molecule MyD88. Although Cbl-b seems to be required for TLR4 ubiquitination under resting conditions, down regulation of TLR4 after activation was not due to Cbl-b-mediated ubiquitination and degradation (127). Later, it was shown that Cbl-b regulates LPS-induced TLR4 signaling by ubiquitination of the adaptor molecules MyD88 and TRIF (120). Stimulation of TLR4 with saturated fatty acids, however, induced ubiquitination and degradation of TLR4 in a Cbl-b dependant manner without ubiquitination of MyD88 or TRIF (125). Thus, Cbl-b is involved in the control of TLR4-mediated activation of myeloid cells through different mechanisms depending on the actual stimulus.

Toll-like receptor-triggered responses are regulated through several negative feedback loops. Signaling through the integrin CD11b activates Syk leading to degradation of the TLR adaptor molecules MyD88 and TRIF by Cbl-b. For this process, the E3 ligase activity is required (120). In contrast, another integrin-mediated pathway to dampen TLR induced activation is independent of this Cbl-b/MyD88 regulatory axis (134). Adhesion to endothelial cells is an important step in the recruitment of mononuclear phagocytes to inflamed tissue. Of the various adhesion molecule interactions involved, association of LFA-1 with ICAM-1 but not VLA-4-mediated adhesion is regulated by Cbl-b. *Cblb-*deficient cells showed increased association of the LFA-1 β chain with 14-3-3β, one of the cytoplasmic factors transmitting inside-out activating stimuli (135).

Using bone marrow-derived mast cells from mice and the rat basophilic leukemia cell line RBL-2H3 quite some information has been gathered on the role of Cbl-b in mast cells. While both c-Cbl and Cbl-b are expressed in rodent mast cells and become tyrosine phosphorylated upon FcεRI engagement (136) for human cells only data on c-cbl exist. In the rodent models, Cbl-b but not c-Cbl has been associated with reduced phosphorylation of various proteins involved in the signaling cascade such as Syk (136, 137), resulting in a negative regulation of FcRεI-induced degranulation (136) and cytokine production (138). Mice genetically engineered to express Cbl-b with a dysfunctional RF domain revealed increased phosphorylation of tyrosine kinases Syk and Lyn and downstream substrates such as LAT (linker for activation of T cells) as well as PLCγ1 (91), similar to that found in mast cells of Cbl-b-deficient mice indicating that the E3 ligase activity is necessary. However, downstream effects seemed to be differentially affected. Phosphorylation of IKKα/β and cytokine production were much stronger in cells where the complete Cbl-b protein was missing compared to cells with a Cbl-b protein mutated in the RF. This shows that the regulatory function of Cbl-b for IgE-induced inflammatory cytokine production is largely independent of the RF domain (91).

Cbl-b is also involved in the regulation of osteoclast activity as *cblb-*deficient mice display osteopenia (139). It was later shown that bone resorption is predominantly regulated by Cbl-b, whereas osteoclast survival is dependent on interaction between c-Cbl and PI3K (140). Thus, Cbl-b may also serve as potential target to modify bone metabolism in diseases such as osteoporosis.

## Cbl-b in Autoimmunity

As genetic ablation of *cblb* is associated with spontaneous development of autoimmunity and increased susceptibility to experimental induction of autoimmune diseases (4, 5) it is not surprising that several groups later found links between genetic *cblb* variants and susceptibility to autoimmunity in an animal model for diabetes (141) as well as in various human autoimmune diseases such as type 1 diabetes (142), lupus erythematodes (143), asthma (144), and multiple sclerosis (145–147). Some of the single nucleotide polymorphisms have been found in the promoter region, possibly influencing transcriptional regulation. Indeed, reduced expression of Cbl-b in PBMC, or more specifically in CD4^+^ T cells, was a common finding in several autoimmune diseases (143, 148–150) and *CBLB* mRNA levels in T cells were inversely correlated with relapse rates in multiple sclerosis patients (151). Consistent with reduced Cbl-b levels, several studies also demonstrated increased proliferation of T cells from autoimmune patients especially under anergic conditions (149, 150) and reduced susceptibility to inhibition by Treg (148). A recent study demonstrated that the transcription factor C/EBPβ displayed stronger binding to a disease associated variant and that the negative effect of IFN-β stimulation on T cell proliferation was compromised in *CBLB* risk allele carriers (150). The functional consequences of other variants, especially in the coding region of *CBLB* still await elucidation.

## Improving Cancer Immunotherapy by Targeting Cbl-b in Clinical Settings

Translation of Cbl-b targeting to clinically applicable concepts requires transient and reversible inhibition of Cbl-b activity. Therefore, we established a synthetic siRNA transfection protocol and demonstrated that transient siRNA-mediated *cblb* silencing results in hyperresponsiveness, decreased TGF-β sensitivity, and increased IFN-γ production (122). Repeated adoptive cell therapy of *cblb* silenced T cells in combination with the DC vaccine resulted in delayed tumor outgrowth and prolonged survival rates without overt signs of autoimmunity. In agreement with the phenotype of *cblb*-deficient murine CD8^+^ T cells, siRNA-mediated knockdown of *CBLB* in naive human CD8^+^ T cells similarly shows markedly enhanced IFN-γ production, even in the absence of CD28 co-stimulation. *CBLB* mRNA was almost undetectable for 1 week suggesting that nucleofected T cells should stay hyperreactive for several days within the patient upon adoptive transfer (122, 152). We here envision using *CBLB* silenced autologous cells as combination partner for other immune activating therapies, such as checkpoint monoclonal antibodies, DNA-based vaccines, and DC vaccination (153, 154).

Adoptive cell therapies have achieved promising results in clinical trials. *Ex vivo* expanded tumor-infiltrating lymphocytes (TILs) have induced regression in patients with metastatic melanoma (155, 156) and virus-specific T cells are used in virus-associated hematologic malignancies (157). Efficacy of adoptive immunotherapy can be increased by *ex vivo* gene transfer in order to bypass the need of the *ex vivo* expansion and accumulation of tumor-specific T cells prior the ACT (158–160). Nevertheless, although tumor-specific immune responses are often induced, the therapeutic efficacy of these approaches is limited because of insufficient *in vivo* activation, expansion, and survival of transferred effector immune cells (159). In addition, the restricted knowledge of tumor antigens that are capable to induce potent anti-tumor immune responses limits the applicability of ACT therapies so far. Besides the unsatisfactory clinical benefit, another disadvantage of common ACT approaches is that they generally require time- and cost-consuming protocols to generate enough tumor-reactive CD8^+^ T cells. Moreover, the use of lentiviral vectors to generate TCR-transgenic T cells harbors the risk of insertional mutagenesis, potentially causing leukemogenesis (161). Therefore, using hyperreactive polyclonal non-TCR-transgenic T cells instead of tumor-specific cells for ACT in combination with a second stimulus as, for example, DC vaccination would simplify the standard operating procedure. Strategies rendering adoptively transferred effector cells resistant to inhibitory cues from the microenvironment are highly attractive for improvement of the efficacy of cancer immunotherapy. Our results obtained in preclinical mouse models suggest that transient siRNA-mediated *CBLB t*argeting in polyclonal CD8^+^ T cells may improve adoptive T cell therapy in the clinic (74, 122, 152). Moreover, decreasing Cbl-b expression of NKT cells stimulated by α-galactosylceramide for ACT (162) may render them resistant to anergy (163) thus improving ACT (164). Alternatively, other stimulating ligands, which do not up-regulate Cbl-b in NKT cells may be useful in the clinical setting (163). As recently shown, also NK cell-based immunotherapies are amenable to improvement by modulation of Cbl-b. Adoptive transfer of *cbl-b* deficient NK cells significantly reduced tumor burden in a melanoma mouse model (89). Similar effects can also be achieved by inhibiting the most prominent targets of Cbl-b ubiquitination in NK cells, the TAM receptor kinases with small molecule compounds.

## Final Comments

Although relatively little information is available regarding the mechanisms of Cbl-b in human cells ample evidence indicates that Cbl-b exerts important regulatory functions in diverse cells of hematopoietic origin, resulting in a balanced immune response. Depending on the clinical situation, either inhibiting or strengthening Cbl-b functions might be desirable. In the autoimmune setting, inhibition of degradation of Cbl-b interaction partners by proteasome inhibitors could constitute a rationale approach (1). Vice versa, anti-cancer immunity could be enhanced by direct or indirect inhibition of Cbl-b function. Small molecule inhibitors specific for Cbl-b have not yet been developed. However, inhibition of molecular substrates of Cbl-b ubiquitination such as TAM receptors that successfully increased NK cell-mediated cytotoxicity and reduced metastatic spread are promising candidates to be tested in clinical trials (89). Also, the recent promising results of clinical studies with immune checkpoint antibodies may be based on Cbl-b. Another way to harness this regulatory pathway for reinforcing tumor-specific cells is to down modulate Cbl-b by molecular means (e.g., by siRNA) for adoptive cellular immunotherapy. The feasibility of such an approach will soon be investigated in a phase I clinical study (NCT02166255). In summary, targeting the immunological gate keeper Cbl-b opens new avenues to treat human diseases, such as cancer and autoimmunity.

## Conflict of Interest Statement

The authors declare that the manuscript was prepared in the absence of any conflict of interest. Dominik Wolf is co-applicant of the patents WO/2009/073905 and WO/2010/119061 A1 related to Cbl-b.

## Abbreviations

ACT, adoptive cell transfer; AP-1, activator protein-1; APC, antigen presenting cell; BCR, B cell receptor; BLNK, B cell linker protein (=SLP-65, BASH); BMDC, bone marrow derived dendritic cell; BTK, Bruton’s tyrosine kinase; Cbl-b, Casitas B-lineage lymphoma proto-oncogene B; C/EBP-b, CCAAT-enhancer binding protein beta; CIN85, Cbl-interacting protein of 85 kDa; CTLA-4, cytotoxic T-lymphocyte antigen 4; DC, dendritic cell; DEC-205, CD205; EAE, experimental autoimmune encephalomyelitis; FOX, forkhead box; Fyn, proto-oncogene; ICAM-1, intercellular adhesion molecule 1; IFN-γ, interferon gamma; IKK α/β, inhibitor of NF-κB kinase alpha/beta; IL-2, interleukin-2; KIR, killer-Ig-like receptor; LAT, linker for activation of T cells; Lck, lymphocyte specific tyrosine kinase; LCMV, lymphocytic choriomeningitis virus; LFA-1, leukocyte function-associated antigen 1; LPS, lipopolysaccharide; MAPK, mitogen-activated protein kinase; MICA, MHC I polypeptide related sequence A; MIP-1, macrophage inflammatory protein-1; MCP-1, monocyte chemoattractant protein-1; mTOR, mammalian target of rapamycin; MyD88, myeloid differentiation primary response gene; Nedd4, neural precursor cell expressed developmentally down-regulated protein 4; NFAT, nuclear factor of activated T cells; NF-κB, nuclear factor of κB; NKG2D, killer cell lectin-like receptor; OVA, ovalbumin; PAMP, pathogen-associated molecular patterns; PD1, programed cell death 1; PD-L1, programed cell death ligand 1; PKCθ, protein kinase C-theta; PI3K, phosphatidylinositol 3-kinase; PLCγ, phospholipase C-gamma; PTEN, phosphatase and tensin homolog; PTK, phospho tyrosine kinase; Rac, member of Rho family GTPases; *rag2*, recombination activating gene-2; RANK, receptor activator of NF-κB; RANTES, regulated on activation, normal T cell expressed and secreted; Ras, member of family of small GTPases; RING, really interesting new gene; SH2, Src homology domain; SIINFEKL, OVA_257–264_ peptide; SLP76, SH2 domain containing leukocyte protein of 76kD; SMAD7, mothers against decapentaplegic homolog 7; Src, proto-oncogene Src; TAM, tyro3, axel, mer family of TAM receptor tyrosine kinases; TCR, T cell receptor; TNF-α, tumor necrosis factor-alpha; TKB, tyrosine kinase binding domain; TRAF, TNF receptor-associated factor; TRIF, TIR-domain containing adapter; TGF-β, transforming growth factor-beta; TLR, toll-like receptor; Treg, regulatory T cell; TRK, receptor tyrosine kinase; Ub, ubiquitin; UBA, ubiquitin-associated domain; Vav1, protooncogene Vav1; VLA-4, very late antigen 4; WASP, Wiskott–Aldrich syndrome protein; Wt, wildtype; ZAP-70, zeta-associated protein of 70 kDa.
